# Role of EBUS-TBNA in Non-Neoplastic Mediastinal Lymphadenopathy: Review of Literature

**DOI:** 10.3390/diagnostics12020512

**Published:** 2022-02-16

**Authors:** Valentina Scano, Alessandro Giuseppe Fois, Andrea Manca, Francesca Balata, Angelo Zinellu, Carla Chessa, Pietro Pirina, Panos Paliogiannis

**Affiliations:** 1 Department of Medical, Surgical and Experimental Sciences, University of Sassari, 07100 Sassari, Italy; agfois@uniss.it (A.G.F.); amanca989@gmail.com (A.M.); francesca.balata90@gmail.com (F.B.); pirina@uniss.it (P.P.); ppaliogiannis@uniss.it (P.P.); 2 Unit of Respiratory Diseases, University Hospital Sassari (AOU), 07100 Sassari, Italy; 3 Department of Biomedical Sciences, University of Sassari, 07100 Sassari, Italy; azinellu@uniss.it; 4 Postgraduate School in Hospital Pharmacy, University of Sassari, 07100 Sassari, Italy; c.chessa@studenti.uniss.it

**Keywords:** EBUS-TBNA, mediastinal lymphadenopathy, granulomatous diseases

## Abstract

Mediastinal lymphadenopathy is a condition in which one or more mediastinal lymph nodes are enlarged for malignant or benign causes, generally more than 10 mm. For a long time, the only way to approach the mediastinum was surgery, while in last decades endoscopic techniques gained their role in neoplastic diseases. At the present time, EBUS is the technique of choice for studying the mediastinum in the suspicion of cancer, while there are not strong indications in guidelines for the study of benign mediastinal lymphadenopathy. We reviewed the literature, looking for evidence of the role of EBUS in the diagnostics of non-neoplastic mediastinal lymphadenopathy, with special regard for granulomatous disease, both infectious and non-infectious. EBUS is a reliable alternative to surgery in non-neoplastic mediastinal lymphadenopathy, even if more evidence is needed for granulomatous diseases other than tuberculosis and sarcoidosis.

## 1. Introduction

The term “lymphadenopathy” refers to a condition in which one or more lymph nodes are enlarged for a known or unknown cause, generally for the hyperplasia of one or more cell types [1]. Studying mediastinal lymph nodes has always been challenging because of their position and the relative difficulty in reaching the mediastinal structure. Formerly surgery was the only way to access them: the main surgical procedures used were parasternal mediastinotomy and some mini-invasive surgical procedures such as extended cervical mediastinoscopy (ECM), video-assisted mediastinoscopy (VAM), and video-assisted thoracoscopic surgery (VATS) [2,3]. However, mediastinal surgery is burdened by some potential life-threatening complications, such as bleeding, mediastinitis, pneumothorax, and/or subcutaneous emphysema, and rarely damage to adjacent organs [2]. 

Between the 1980s and 1990s, the endoscopic study of the mediastinum was studied and developed, which gained progressive importance for its minimal invasiveness and a minor rate of complications compared with surgery, in addition to a good accuracy. Endoscopic mediastinal procedures using ultrasound are represented by endoscopic (transoesophageal) ultrasound (EUS) and endobronchial ultrasound (EBUS) [2]. 

The main aims in studying mediastinal lymph nodes are the diagnosis and staging of malignancies, especially lymphomas and non-small cell lung cancer (NSCLCs), as well as metastases from other organs’ tumours (oesophago-gastric cancer [4,5], thyroid cancer [6], seminoma [7], breast cancer [8]). 

Mediastinal lymph nodes’ alterations may also be due to benign diseases, including infectious or inflammatory diseases such as tuberculosis or sarcoidosis, which are the principal causes. The purpose of the present review is to examine the role and the diagnostic yield of endobronchial ultrasound in studying benign mediastinal lymphadenopathy.

## 2. Mediastinal Lymph Nodes Stations

**Figure 1:**
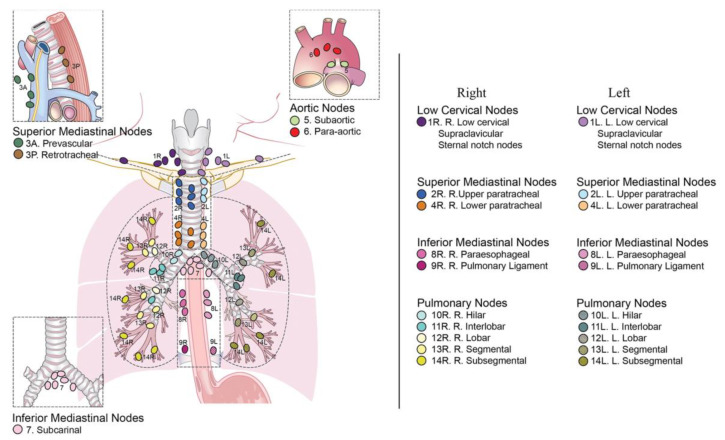
IASLC mediastinal lymph node map, courtesy of El-Sherief et al. [11].

The mediastinum is an anatomic compartment placed at the middle of the thoracic cavity, extending from diaphragm to superior thoracic inlet in continuity with neck structures, inside which are included all the thoracic organs except for lungs and pleurae. There is not a universally accepted partition; however, more often it is divided in three parts named anterior, middle, and posterior mediastinum. Some authors identify also a “superior” mediastinum. The anterior or prevascular mediastinum extends from the superior thoracic inlet to the site of connection of the heart with the thoracic wall; the middle mediastinum is the space containing the heart and great vessels; the posterior or postvascular mediastinum is delimited anteriorly by the posterior face of the pericardium and great vessels and posteriorly by the anterior face of the vertebral bodies. The superior mediastinum may be defined as the region above an imaginary line extending from the sternal angle or above the aortic arch [9,10]. All of these compartments contain lymph nodes. A nodal map is provided by the International Association for the Study of Lung Cancer (IASLC) [11], which divides mediastinal lymph nodes into fourteen stations, classified by their anatomical position. A “R” or a “L” is added to specify whether they are on the right or on the left side, respectively, as well as “A” and “P” are for “anterior” or “posterior” (Figure 1).

- Supraclavicular region contains the supraclavicular, low cervical, and sternal notch lymph nodes, named stations 1R and 1L;
- Upper zone (superior mediastinum region) contains the right and left upper paratracheal lymph nodes (stations 2R and 2L), prevascular lymph nodes (station 3A), retrotracheal lymph nodes (station 3P), and right and left paratracheal lymph nodes (stations 4R and 4L);
- Aortopulmonary zone contains the subaortic (station 5) and paraaortic (station 6) lymph nodes;
- Subcarinal zone contains the subcarinal lymph node (station 7);
- Lower zone contains the paraesophageal (station 8) and pulmonary ligament (station 9) lymph nodes;
- Hilar and interlobar zone contains the hilar (station 10) and interlobar (station 11) lymph nodes;
- Peripheral zone refers to pulmonary nodes, and includes the lobar (station 12), segmental (station 13), and subsegmental (station 14) lymph nodes.

All of the lymph node stations described may be studied with both non-invasive and invasive or minimally invasive techniques. 

## 3. Studying Mediastinal Lymph Nodes

### 3.1. Non-Invasive Techniques

Non-invasive techniques are represented by imaging studies.

#### 3.1.1. Chest Radiography

Chest radiography is a less sensitive exam. It can identify lymph node enlargement when it causes an alteration in the mediastinal contour, but it must be completed with further exams. A normal chest radiograph in a patient with signs of mediastinal mass or suspected of a disease involving the mediastinum does not exclude diagnosis, and so it should be avoided as a first-line exam [12].

#### 3.1.2. CT

Computed tomography (CT) has dramatically changed the non-invasive study of mediastinal lymph nodes. Compared with chest radiography, it allows precise evaluation of the size, shape, extension, localization, and relation with adjacent organs [13]. The diameter of most of thoracic lymph nodes is less than 10 mm, and when this diameter is increased, a neoplastic or inflammatory/infective disease must be excluded, even if lymphadenopathy may be subsequent also to pulmonary hypertension and cardiac failure [14]. Additionally, the shape may suggest the malignant nature of the lymphadenopathy. Differences in attenuation and density may enhance suspicion of the nature of the lymphadenopathy, but in most cases, it must be confirmed by histological sampling [10].

#### 3.1.3. MRI

Magnetic resonance imaging (MRI) of the mediastinum allows the avoidance of ionizing radiations and the acquisition of multiplanar images, together with the possibility of administering liquid of contrast even in nephropathic patients; nevertheless, the prolonged time for the acquisition of the images, which may be disturbed by artifacts due to cardiac and respiratory movements, and the inferior yield in studying pulmonary parenchyma affects the advantages. Indications of MRI in mediastinal study are limited, and it should probably be reserved only in those patients who cannot undergo CT or in whom post-contrast CT is inconclusive [15].

#### 3.1.4. PET and PET/CT

18-Fluorodesoxyglucose positron emission tomography (^18^FDG-PET) provides information about activity and behaviour of lymph nodes, integrating information about the size and shape provided by CT (PET/CT). However, it is not quite sensitive, for both neoplastic and inflammatory lymph nodes may pick up FDG. As for the CT, the diagnostic yield is impaired by lymph node dimension, with higher rate of false negative exams when lymph node diameter is <10 mm [16].

### 3.2. Endoscopic Minimally Invasive Techniques

**Figure 2:**
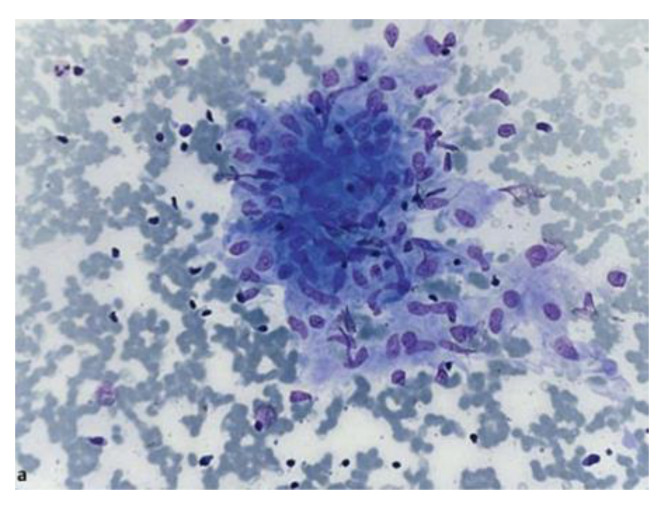
Cytological sample showing loosely aggregated epithelioid cells without necrosis, stained in Diff Quick for rapid on-site evaluation. Courtesy of von Bartheld et al. [37] who own the copyright.

Ultrasound allows the study of the architecture [17], perfusion [18], resistance index, and elasticity of lymph nodes [19]. Nakajima et al. described also a ultrasonographic vascular pattern classification ranging between grade 0, which means no blood flow, to grade 3, which means more than four vessels and/or twist helical flow signal [20]. Endobronchial ultrasound (EBUS) is currently indicated as the first-line exam in staging mediastinal lymph nodes in NSCLC [21,22,23], but several studies demonstrated a sensitivity and specificity equivalent to mediastinoscopy in the evaluation of thoracic lymph nodes enlargement [24,25,26,27]; the positive rate seems to decrease in the elderly, but with maintained specimen quality and safety [28]. Its diagnostic yield is enhanced by ancillary techniques such as rapid onsite evaluation (ROSE), which should evaluate the sample for the presence of lymphocytes and material from lesion [27,29]; Plit et al. demonstrated a positive predictive value of 97.7% for EBUS-TBNA plus ROSE in diagnosing sarcoidosis [30]. Many other randomized trials compared in a meta-analysis demonstrated no increase in diagnostic accuracy with ROSE [26], so at the present time, guidelines do not suggest its use after every procedure; however, it can reduce complications by reducing the number of passages needed for diagnosis [31,32,33,34]. Furthermore, there is evidence that a trained pulmonologist can correctly assess the material obtained [35]. Cell block, another ancillary technique, may improve the diagnostic yield of EBUS-TBNA in sarcoidosis [36]. In Figure 2 is shown a granuloma sampled with EBUS-TBNA and stained with Diff Quick for rapid on-site evaluation.

EBUS allows cytological or histological samples (for example via mini-forceps [38]) to be reached, evaluated, and obtained from central masses or lymph nodes localized near the trachea and principal bronchi. It can be realized with radial or longitudinal (convex) probes. Radial probes provide a 360-degree view, with higher definition of the layers of the bronchial wall, thus also allowing evaluation of an eventual infiltration of the structures. Radial probes (which may be classified also in central and peripheral probes on the base of the MHz) are placed within the operative channel of a flexible or rigid bronchoscope inside a guide-sheath. However, a radial probe does not allow real-time sampling. Longitudinal probes allow real-time transbronchial needle aspiration (TBNA): they are placed on the tip of the bronchoscope and provide a 90-degree angled view of what is parallel to the bronchoscope shaft. Usually, they use a frequency of 7–7.5 MHz; Doppler and elastography may be implemented. Some authors suggest sampling each lymph node at least twice [39,40], while others at least thrice [27,36,41].

**Figure 3:**
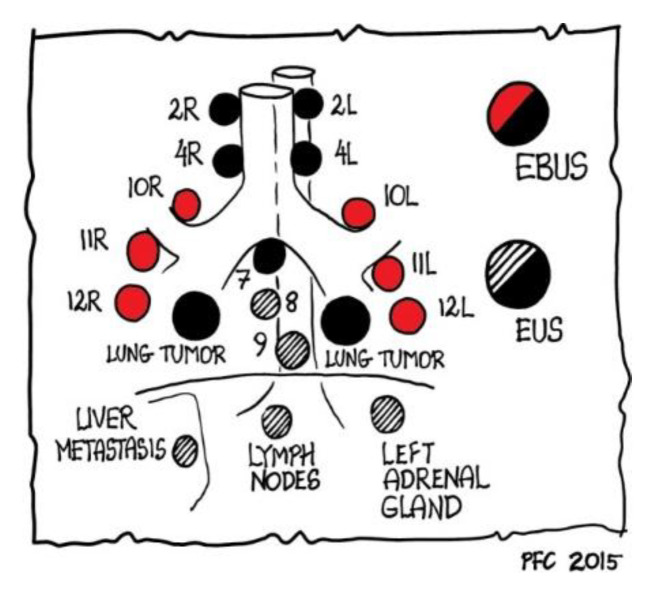
Role of EBUS and EUS in reaching mediastinal lymph nodes, as outlined by Paul Clementsen [2] who is the owner of the copyright.

Endoscopic ultrasound (EUS) is performed via transoesophageal access and allows central masses and lymph node sampling with a fine needle aspiration technique. Its role is complementary to EBUS because it can sample lymph node stations localized far from the carina (stations 8 and 9); also, via EUS it is possible to reach adrenal gland metastasis (Figure 3).

The two of them are efficient and safe: the diagnostic yield in investigating a generic mediastinal lymphadenopathy has been calculated to be 88% for EUS-FNA and 92% for EBUS-TBNA [42], with a mortality rate inferior to 1% (0% for EUS-FNA and 0–0.8% for EBUS-TBNA, individually); Santos et al. reported a general diagnostic yield for EBUS-TBNA in isolated mediastinal lymphadenopathy of 77.6% [43]. In addition, the morbidity rate is 0–2.3% for EUS-FNA and 0–1.2% for EBUS-TBNA [2,42,44]. Kuo et al. demonstrated on a sample of 83 patients who underwent EBUS-TBNA a very similar diagnostic yield for malignant and non-malignant diseases [39,45]. Independent predictors of better predictive value are short diameter of lymph node >16.5 millimetres and sampling mediastinal lymph nodes rather than hilar ones [39]. Among granulomatous diseases, the diagnostic accuracy of EBUS-TBNA is reported to range between 74.5% and 96% [39,46,47,48,49,50]. The sensitivity, diagnostic accuracy, and negative predictive value of EBUS-TBNA in patients with a previous negative conventional TBNA are 87.8%, 90.1%, and 65.7%, respectively [51]. Shen et al. showed that EBUS-TBNA compared with conventional TBNA has a greater diagnostic yield both for granulomatous and non-granulomatous benign lymphadenopathy, especially when the diameter of the lymph node sampled was lower than 20 mm [52]. In a single centre study conducted on 100 patients, the use of EBUS-transbronchial fine needle biopsy (TBNB) with Fransen needle had a diagnostic yield of 97% [53].

### 3.3. Surgical Techniques

As previously stated, the mediastinum can be surgically explored principally with parasternal mediastinotomy (Chamberlain procedure), mediastinoscopy (both cervical extended or video-assisted), and video-assisted thoracoscopic surgery. Surgery is currently reserved for those cases in which an endoscopic procedure is not indicated or does not provide a diagnosis, and furthermore, surgery may also provide a therapeutic intent by removing lymph nodes [2].

## 4. Causes of Mediastinal Lymphadenopathy

Mediastinal lymph nodes enlargement with short axis diameter >10 mm is conventionally defined a mediastinal lymphadenopathy. Some authors suggest that the normal short axis diameter may vary between nodal stations: in particular, 10 mm is the limit for station 4 lymph nodes, while for station 7 lymph nodes it is 12 mm, and for the other stations it is 8 mm [54]. The causes of mediastinal lymphadenopathy may be summarized as:

1. Malignant causes, principally represented by neoplastic causes.
2. Benign causes, represented by infectious, inflammatory, and reactive causes.

A resumptive scheme of the principal causes of mediastinal lymph node enlargement is shown in Table 1. 

A neoplastic involvement of mediastinal lymph nodes is generally due to NSCLCs’ metastasis or haematological malignancies, such as lymphomas, myeloma, or leukaemia. Nodal metastasis may come from a gastrointestinal or oesophageal tumour, as well as from a tumour of the breast, testicle, or thyroid, as previously reported. 

Numerous infective pathogens may cause mediastinal lymph node enlargement: the major responsible pathogen worldwide is *Mycobacterium* (both *M. tuberculosis* and atypical *M*.), but also fungi (*Coccidioides*, *Histoplasma*), other bacteria (*F. tularensis*, *B. anthracis*), and viruses (HIV, Epstein–Barr virus). Based on the type of material obtained by fine needle aspiration (FNA), infective lymphadenopathy can be further classified as suppurative and granulomatous; different stains are indicated for each type [55].

Inflammatory causes include sarcoidosis, rheumatological and autoimmune diseases (e.g., SLE, rheumatoid arthritis, systemic sclerosis), cystic fibrosis, pneumoconiosis, hypersensitivity pneumonia, amyloidosis, Whipple disease, Rosai–Dorfman disease, and Castleman’s disease.

In the end, a (usually temporary) lymphadenopathy may appear during pneumonia or in case of pulmonary oedema, as well as in pulmonary hypertension, idiopathic pulmonary fibrosis (IPF), COPD, and chronic heart failure [56]. Many drugs are associated with lymph node enlargement secondary to a hypersensitivity reaction [57]. Anecdotally, a granulomatous mediastinal nodal involvement secondary to silicone breast implant has been described [58].

Since it was principally developed for this purpose, the role of EBUS in neoplastic disease, most of all NSCLC, is extensively treated in the literature. Conversely, its role in investigating benign causes of lymphadenopathy is less known. The aim of the present review is to focus on the principal benign causes of mediastinal lymphadenopathy.

### 4.1. Non-Infectious Causes

#### 4.1.1. Sarcoidosis

Sarcoidosis is one of the most frequent causes of mediastinal granulomatous lymphadenopathy in non-developing countries, although a study from Kuo et al. demonstrated its prevalence in intrathoracic lymphadenopathy without pulmonary lesion also in a TB-endemic country [45]. The cause is unknown, even though many studies suggest a role for *Mycobacteria* and *Propionibacterium* [59]. Immunopathogenesis is not fully understood but seems to imply a role for T-cell receptors and HLA dysfunction in response to exposition to an unknown antigen [59]. Diagnosis requires clinical and radiological criteria, evidence of non-caseating granuloma on histopathology, and exclusion of other granulomatous diseases such as tuberculosis and lymphoma [60]. A radiological classification (Scadding) identifies five stages: lymphadenopathy is seen in stage 1 (lymphadenopathy alone) and stage 2 (lymphadenopathy with parenchymal infiltration) [60]. In these stages, the typical imaging finding is a bilateral, symmetrical perivascular cluster of lymph nodes with a tropism for pleura (pleural avidity). The enlargement of right paratracheal and bilateral hilar lymph nodes is known as Garland’s sign. Although in the ATS/ERS guidelines transbronchial lung biopsy is the diagnostic procedure of choice, its importance has decreased in recent decades thanks to the development of endobronchial ultrasound techniques [61]. Nodal ultrasound findings are represented by a mixed echogenicity, but no change in architectural structure should be seen when using CEUS and Doppler, and the hilum is conserved [41,62,63]. Elastography may help to identify the lymph nodes involved, which usually appear with a blue pattern, meaning an increased stiffness [64]. If conventional “blind” TBNA fails to diagnose sarcoidosis in one third or half of the cases, EBUS-TBNA and EUS-FNA show a sensitivity of 80–90% [46,65,66,67] with a diagnostic yield on 2097 patients of 79% [68]; Ortakoylu et al. report a diagnostic accuracy of 86% [39]. Pooled sensitivity for EBUS-TBNA is reported to be 84% and pooled specificity around 100% [68]. The diagnostic accuracy is around 90% even for Scadding stage 1 [37,45]. A summary of diagnostic yield of EBUS-TBNA in sarcoidosis is shown in Table 2. 

Diagnostic yield seems to be increased by ancillary methods such as liquid-based cytology and cell block [37,69]. In EBUS-guided procedures, neither lymph node location, lymph node size, needle size, nor number of aspirates for node seem to alter sensitivity and specificity [69]. However, endoscopist experience may alter the diagnostic yield: for Pedro et al., 37–44 procedures are required to achieve an accuracy of 80% [61]. A common blood test such as red-cell distribution width (RDW) has been shown to be significantly higher in stage II sarcoidosis compared with stage I sarcoidosis and tubercular lymphadenitis [70]. Furthermore, the analysis of the transcriptional profile on the sample can help to discriminate sarcoidosis from other granulomatous diseases [71]. 

The main complication described is mediastinitis with abscess formation after EUS [37], while 13% of the complications following EBUS with 8–10 passages were reported in one RCT [30]. 

#### 4.1.2. Sarcoid like Reaction (SLR)

In some patients affected with malignancy, a mediastinal lymphadenopathy secondary to a noncaseating granulomatous lymph node involvement may occur: it is named a sarcoid-like reaction (SLR), and it is considered a paraneoplastic manifestation, but it may appear even after chemotherapy or radiotherapy [72]. The cause is yet unknown: Urbanski et al. suggested an “antigen shedding” from cancer as being responsible [73]. The tumour more often associated with SLR is testicular germ cells cancer [73,74], but it is also described to have an association with NSCLC [75]. EBUS-TBNA is useful in settling a differential diagnosis between SLR and metastasis [76].

#### 4.1.3. Silicosis

Silicosis is caused by inhalation of free silica particles, which cause the formation of noncaseating granulomas. Hilar nodal enlargement may precede parenchymal involvement [77], and it is present in 74% of patients [78]. Lymph nodes usually show calcification with eggshell or punctate distribution [78]; under polarized light, histological and cytological samples show nodules of fibroblasts and histiocytes in addition to birefringent particles [79]. In a small multicentre study, Shitrit et al. demonstrated specificity of 100% and sensitivity of 88% for EBUS-TBNA in diagnosing silicosis in patients with consistent exposure history [80].

#### 4.1.4. Berylliosis

Berylliosis is caused by exposure to beryllium compounds. Up to 25% of cases may show a nodal enlargement and noncaseating granulomas similar to sarcoidosis, usually in association with parenchymal fibrosing alterations [81]. Currently there are no studies on diagnostic yield of EBUS in this disease.

#### 4.1.5. Amyloidosis

Amyloidosis is characterized by deposition of abnormally folded fibrillar proteins in lymph nodes and organs and may be classified as being primary or secondary. Bilateral mediastinal lymph nodes enlargement, sometimes with calcification, is most typical of the primary form and may resemble sarcoidosis [82]. Many case reports show a role for EBUS-TBNA in diagnosis [83,84,85].

#### 4.1.6. Castleman’s Disease

It is an uncommon idiopathic lymphoproliferative disease, which may be unifocal (a mediastinal or hilar mass rather than the enlargement of a single nodal compartment) or, more frequently in HIV patients, multifocal. Lymph nodes’ architecture is maintained and during CEUS they gain contrast homogeneously; vascularization is usually increased [86]. Nodal sampling is necessary for establishing the diagnosis.

#### 4.1.7. Other Non-Infectious Causes

Mediastinal lymph node enlargement with maintained architecture may be observed in patients affected with COPD, IPF, and chronic heart failure, probably secondary to pulmonary hypertension and/or diffuse intrathoracic oedema [87]. In chronic left heart failure, the lymph nodes more often enlarged are the hilar, subcarinal, and paratracheal ones [57]. 

Up to 68% [88] of IPF patients show mediastinal lymphadenopathy, which has a correlation with disease severity and progression risk [89]. Furthermore, they have a higher risk of developing lung cancer, so in IPF patients, EBUS has a major role in early lymph node evaluation during follow-up [90].

Half of COPD patients have enlarged lymph nodes, especially those showing a bronchitic phenotype [91]. The lymph nodes involved, usually oval-shaped and showing signs of reactive enlargement, are those in the aorto-pulmonary windows, the paratracheal ones, and the subcarinal ones [57].

### 4.2. Infectious Causes

#### 4.2.1. Mycobacterial Diseases

*Mycobacterium tuberculosis* or Koch bacillus and other *Mycobacteria* usually cause the formation of granulomas in lung and lymph nodes. Tubercular lymphadenopathy is the most frequent extrapulmonary manifestation of tuberculosis, especially in the primary subtype [92]. In countries where it is still endemic, in patients showing mediastinal lymphadenopathy, a mycobacterial disease should be excluded [93,94], in particular in those patients without clear home exposure to tubercular patients [45]. Clinical presentation may be similar to sarcoidosis, and so the imaging features. Microbiological exams (Ziehl–Neelsen stain, polymerase chain reaction, microbiological culture) on sputum are diagnostic, but also show also low sensitivity; additionally, sarcoidotic granuloma sometimes may also show necrosis [95]. In recent decades, EBUS-TBNA has been gaining importance in the differential diagnosis between those two granulomatous diseases [96,97]. The diagnosis is based on the presence of epithelioid granulomas with or without caseous necrosis associated with presence of *Mycobacterium* in the same sample or sputum treated with appropriate stain (acid-fast bacilli or Ziehl–Neelsen stain) [55]. Culture is the gold standard for the diagnosis, and together with microscopic exam reaches a specificity of 95% [55]. A positive tuberculin skin reaction supports the diagnosis. The overall sensitivity, predictive negative value, and diagnostic accuracy range between 64% and 81%, 33% and 43%, and 70% and 83%, respectively [98,99] (Table 3). The sensitivity of EBUS-TBNA in diagnosing tuberculosis is reported to be 74–85% [39,100]. TB-PCR on the sample increases the diagnostic rate [101], with a sensitivity of 56%, specificity and positive predictive value of 100%, negative predictive value of 81%, and diagnostic rate of 85–96% [102,103]. 

EBUS can be useful in suspected tubercular lymphadenitis, also with a simple sonographic evaluation of lymph nodes: in tuberculosis, the echogenicity of lymph node is more often heterogeneous, and necrosis signs are more frequent [40,57]. 

EBUS may have a therapeutic role in the drainage of tubercular abscesses, a rare complication of colliquation of multiple lymph nodes [104].

#### 4.2.2. Fungal Diseases

Fungal nodal involvement seems to be associated more frequently with caseous granulomas, and thus in endemic areas or whether there is high suspicion of fungal disease, an appropriate stain should be applied in samples obtained [105]. The most frequent fungal infections associated with mediastinal lymphadenopathy are cryptococcosis, histoplasmosis, and coccidioidomycosis [57]. Cryptococcus neoformans not only may cause pneumonia but also may cause disseminated infections with lymph node involvement, especially in immunocompromised hosts. Typical findings are neutrophils associated with some lymphocytes, granulomas, and histiocytes and yeasts surrounded by a “halo” capsule [55]. 

Histoplasmosis is a systemic infection caused by the inhalation of spores of Histoplasma capsulatum; in the acute form, it may be associated with enlarged mediastinal lymph nodes with central necrosis or colliquation, surrounded by a fibrous capsule. Granulomas may be seen as well [57]. Loosely formed granulomas may be observed in subacute pulmonary histoplasmosis [106]. However, rarely is the culture positive on the sample obtained by EBUS-TBNA, and the diagnosis is aided by infectious serologies and antigen testing [106]. 

Coccidioidomycosis is caused by the inhalation of spores of Coccidioides sp. Mediastinal lymphadenopathy is most typical of the acute form of the disease and usually correlates with a systemic dissemination [107]. Lymph node enlargement may persist even after resolution of the parenchymal lesions [108].

#### 4.2.3. Viral Diseases

Mediastinal lymphadenopathy may appear during viral illnesses, but it is not common and, when present, it is associated with pneumonia or, as it happens in Epstein–Barr virus infection, together with systemic lymph nodes enlargement [109]. It is not specific and more often resolves after healing. 

In HIV patients showing mediastinal lymphadenopathy, EBUS-TBNA is useful both to exclude an infection and a lymphoma degeneration or Kaposi’s sarcoma, which sometimes may occur with only visceral involvement. Typical cytopathological findings include casually arranged spindle cells which may resemble granulomas [110].

## 5. Conclusions

Despite its main role in the diagnosis and staging of cancer, the literature demonstrates that EBUS-TBNA is a safe and reliable procedure for diagnosing non-neoplastic diseases involving mediastinal lymph nodes, especially sarcoidosis, instead of surgery. More randomized controlled trials are needed to establish its effective diagnostic yield in benign diseases other than sarcoidosis and tuberculosis.

## Figures and Tables

**Table 1:** Scheme of principal causes of mediastinal lymph node enlargement.

Causes of Mediastinal Lymphadenopathy	
Malignant Causes	Benign Causes
**Primary** Haematological malignancies Lymphoma Myeloma Leukaemia	**Infectious** *Aetiology:* **Bacterial** *Mycobacteria* spp. *F. tularensis* *B. anthracis* **Viral** EBV HIV **Fungal** *H. capsulatum* *Coccidioides* spp. *Cytology:* **Suppurative**: *Streptococcus* spp., *Staphylococcus* spp., *Klebsiella* spp., *Candida* spp., *HIV* **Granulomatous**: see above
**Secondary (nodal metastasis)** NSCLCs Gastrointestinal tumours Brest cancer Testicle cancer Thyroid cancer	**Inflammatory** Sarcoidosis Rheumatological and autoimmune diseases Cystic fibrosis Pneumoconiosis Hypersensitivity pneumonia Amyloidosis Whipple disease Rosai–Dorfman disease Castleman’s disease Silicone breast implant
	**Reactive** Pneumonia Idiopathic pulmonary fibrosis COPD Pulmonary oedema Pulmonary hypertension Chronic heart failure Hypersensitivity reaction to drugs

**Table 2:** Resumptive table of diagnostic yield of EBUS-TBNA in sarcoidosis.

Author	EBUS Diagnostic Yield	Population	Type of Study
Tremblay et al. 2009	83.3%	50	Randomized controlled trial
Nakajima et al. 2009	91.4%	35	Randomized controlled trial
Von Bartheld et al. 2010	92% for Scadding stage I, 77% for Scadding stage II	101	Descriptive study
Navani et al. 2011	85% (93% combined with TBB)	39	Descriptive study
von Bartheld et al. 2013	80%	155	Randomized controlled trial
Ortakoylu et al. 2015	83%	159	Descriptive study
Trisolini et al. 2015	79%	2097	Meta-analysis

**Table 3:** A resumptive table of diagnostic yield of EBUS for mycobacterial disease.

Author	EBUS Diagnostic Yield	Population	Type of Study
Çağlayan et al. 2011	84.2%	19	Descriptive study
Sun et al. 2013	90%	41	Descriptive study
Low et al. 2014	70%	18	Descriptive study
